# Transcriptomic response to thermal and salinity stress in introduced and native sympatric *Palaemon* caridean shrimps

**DOI:** 10.1038/s41598-017-13631-6

**Published:** 2017-10-25

**Authors:** Amandine D. Marie, Steve Smith, Andy J. Green, Ciro Rico, Christophe Lejeusne

**Affiliations:** 1 School of Marine Studies, Molecular Analytics Laboratory (MOANA-LAB), Faculty of Science technology and Environment, The University of South Pacific, Laucala Campus, Suva, Fiji; 2 Konrad-Lorenz-Institue of Ethology, Department of Integrative Biology and Evolution, University of Veterinary Medicine, Vienna, Austria; 3 Estación Biológica de Doñana, Consejo Superior de Investigaciones Científicas (EBD- CSIC), Sevilla, 41092 Spain; 4 Sorbonne Universités, UPMC Université Paris 06, CNRS, UMR 7144, Station Biologique de Roscoff, 29688 Roscoff, France; 5 CNRS, UMR 7144, Station Biologique de Roscoff, 29688 Roscoff, France

## Abstract

Organisms develop local adaptations to cope with spatially and temporally variable environments such as estuarine habitats, where abiotic parameters such as salinity and temperature fluctuate continuously. Studying the regulation of gene expression in a variable environment allows us to understand the underlying molecular mechanisms of these adaptations and the relative roles of the genetic and plastic response. The transcriptomes of the European native *Palaemon longirostris* (PL) and the introduced *P. macrodactylus* (PM) shrimps are described and compared after an experiment simulating summer conditions in the Guadalquivir Estuary, Spain. Specimens, collected in the Guadalquivir Estuary, were maintained at a temperature and salinity of 20 °C and 5 ppt for the control, and 30 °C and 15 ppt for the stress treatment. A large amount of differential gene expression was observed: 16,013 and 2,594 for PL and PM respectively. Functionally annotated unigenes revealed some differences, with PL seemingly having to face stronger physiological stress than PM. Thus, PM seems to have greater resistance than PL under conditions of high temperature and salinity. These results constitute a step forward in the understanding of the underlying molecular mechanisms of genetic adaptation of native invertebrates, and alien taxa that have successfully invaded estuaries in temperate regions around the world.

## Introduction

Biological invasions constitute one of the major environmental threats to worldwide biodiversity in the 21^st^ century, forming a “deadly duo” with climate change^1,2^. The intensification of marine transport since the industrial revolution and particularly after World War II has resulted in major invasion events in estuarine and coastal marine ecosystems, which are also heavily impacted by climate change, threatening their biodiversity worldwide^3–6^. Temperature and salinity are two of the most prominent abiotic determinants of the spatial distribution of aquatic species and are important selective forces leading to local adaptation. The combined effects of temperature and salinity on the survival of marine and brackish water organisms have been demonstrated in many crustacean species^7–9^. The physiological tolerance of invasive species to these factors has also been compared to that of natives species^10^. The importance of these factors and the need to consider them jointly were emphasized by Kinne^11,12^, who noted that salinity could modify the effects of temperature and alter the temperature range of many biological processes. In turn, temperature can also modify the effects of salinity. These two abiotic factors generate daily stress for many organisms in estuaries and coastal environments^13^. Organisms living in such ecosystems must adapt^14,15^ to cope with constant fluctuations of salinity and temperature due to tides and freshwater inputs from rivers and rainfall.

The capacity of a non-indigenous-species (NIS) to become established, and potentially invasive, depends among other factors on their physiological tolerance to the environmental stress found in the new environment, which in turn is dependent on its standing genetic diversity and its phenotypic plasticity^16^. From the transcriptome of a species submitted to various temperature exposures, the thermal adaptation pathway selected in response to temperature variation may be identified^17,18^. Similarly, the response to salinity stress under acute hyper- and hypo-haline conditions has allowed the identification of physiological pathways involved in osmoregulation^19–21^. However, most studies generally focus on molecular mechanisms and responses associated with a single environmental variable such as temperature or salinity, and thus do not capture the complexity of interactions experienced by wild populations in highly fluctuating environments such as estuarine zones.

Recent advances in massive parallel sequencing technologies permit the characterisation of the whole transcriptomes of organisms under diverse physiological conditions, opening the possibility to study the machinery of adaptation^17^. Furthermore, these technologies have become rapid and highly cost-effective, and the transcriptomic responses induced by thermal stress^17,20,22,23^ and/or salinity stress^19–21,24,25^ is becoming a major focus of the emerging field of ecological genomics^26^. The NGS technology has clear advantages over existing approaches, revealing the complex dynamics of the transcriptome with a unique level of accuracy and sensitivity^27–29^. Moreover, in the case of non-model organisms^17,22,29^, the continual development of high-throughput sequencing and genotyping platforms, as well as the improvement in bioinformatics tools, facilitates the *de novo* assembly of short reads into annotated transcripts and their subsequent analysis.

*Palaemon macrodactylus* (PM) Rathbun, 1902 is an estuarine caridean shrimp native to East Asia (Japan, Korea and China), which was translocated (most likely by ballast water) to San Francisco Bay in the 1960s. Since the 1990s it has subsequently been reported from Argentina, from the northeastern coast of the USA and from Europe^30–32^ where it is now present from South West Spain to Germany and England, and also in the western Black Sea. In European estuaries (e.g. Gironde, Guadalquivir), PM underwent a fast demographic and geographical expansion only a few years after its introduction^32–34^. Several studies have documented the higher physiological tolerance of some non-indigenous species when compared to their native counterparts^35–37^, which could explain their successful establishment. For example, Lejeusne *et al*.^10^ showed that PM is more tolerant to a rapid increase of temperature than *P. longirostris* (PL), a European native sympatric and even syntopic species present within the Guadalquivir Estuary (SW Spain). PM also consumes less oxygen over a broad range of temperatures and salinities^10^ and under acute stress conditions its mortality rate is lower than that of the native species. However, at high levels of salinity, both species were severely affected, with PM showing a significantly higher mortality rate than the native species^10^. Another broader study on the distribution of shallow marine benthic organisms in South Africa showed that non-indigenous species, regardless of their thermal tolerance, range size and genetic variability, were expanding their range and increasing their abundance, and suggests climate change could foster the spread and abundance of non-indigenous organisms across multiple spatial scales^38^. Thus, in a context of increased species translocation due to more and faster transport vectors (e.g. maritime traffic), understanding the molecular mechanisms of adaptation of species to their new environment is of primary importance.

This study constitutes an initial investigation of the transcriptomic response to heat and salinity stress of the European native caridean shrimp PL as well as its introduced congener PM. Our aim was to identify a large spectrum of genes that are either up or down regulated under normal and acute environmental stress conditions commonly found in the natural populations. We chose to vary temperature and salinity at the same time, to simulate the summer conditions that are encountered by these species in the Guadalquivir Estuary from June to September every year^39^. Specimens, collected from the wild, were maintained under control and treatment conditions *via* the manipulation of temperature and salinity. cDNA libraries corresponding to both treatments were sequenced, and *de novo*-assembled before analysing the differential gene expression upon combined heat and salinity stress. Thus, for the first time, the transcriptomes of these two taxa are described and the genes putatively involved in thermal and salt adaptation identified. This constitutes a first step towards understanding the underlying molecular mechanisms of adaptation of invertebrates that have successfully invaded estuaries in temperate regions around the world.

## Results

### Sequencing and *de novo* assembly

For the control group, a total of 38,660,166 and 48,267,988 raw reads were obtained for PL and PM respectively. For the treatment group, a total of 38,848,734 (PL) and 50,000,000 (PM) raw reads were obtained. Table 1 summarises statistics after trimming and quality filtering as well as details of *de novo* assembly. Thus, a total of 72,573 and 33,274 unigenes were obtained for PL and PM respectively. The length of these unigenes ranged from 300 bp to more than 3000 bp (Supplementary Figure S1).

**Table 1:** Statistics after trimming and quality filtering as well as of *de novo* assembly.

	*P. longirostris*	*P. macrodactylus*
*After trimming and quality filtering*		
Number of clean reads		
- control group	35,357,808	38,963,116
- treatment group	36,588,780	41,092,214
Number of nucleotides		
- control group	3,180,202,720	3,506,680,440
- treatment group	3,292,990,200	3,698,299,260
Average G	46.9%	49.8%
Average C	42.4%	49.2%
*De novo assembly*		
Total number of unigenes	72,573	33,274
Total length of unigenes (nt)	40,780,267	19,319,528
Mean length of unigenes (nt)	562	581
N50	728	768
Distinct clusters	15,631	5,400
Distinct singletons	56,942	27,874

### Functional annotation

Blasting and annotation were successful for 35.96% (26,096) and 43.64% (14,521) of unigenes for PL and PM respectively *via* the database pipeline (the NCBI non-redundant protein database “nr”, the nucleotide database “nt”, the Swiss-Prot database, the clusters of Orthologous Groups database “COG”, the Kyoto Encyclopedia of Genes and Genomes database “KEGG” and the Gene Ontology functional classification “GO”). Blastx and ESTScan analyses also revealed that 31,634 (PL) and 16,665 (PM) unigenes had reliable coding DNA sequences (CDS). 24,608 (PL) and 13,540 (PM) unigenes were derived from Blastx and 7,026 (PL) and 3,125 (PM) unigenes from ESTScan. All annotated and CDS containing unigenes were used to construct a reference database for subsequent identification of differentially expressed genes (DGE analysis).

Using BlastX, ca. 8000–25000 unigenes for PL and ca. 5400–13500 unigenes for PM found homology with sequences from the nr, nt and the Swiss-Prot databases (Table 2a). According to the results from the sequence homology of the nr database for the two species, 64.4% ± 0.9 of unigenes showed a strong homology to known genes (E-value <1.0 E^−15^), whereas 35.7% ± 0.9 of unigenes displayed an E-value distribution ranging from 1.0E^−15^ to 1.0E^−5^ (Supplementary Figure S2a(A) and S2b(A)). 66.0% ± 2.0 of unigenes displayed a similarity higher than 40%, whereas 34.1% ± 2.1 of unigenes showed a similarity ranging from 15% to 40% (Supplementary Figure S2a(B) and S2b(B)). The nearest species to PL and PM in terms of blast hits from the nr database was *Daphnia pulex* (Supplementary Figure S2a(C) and S2b(C)).

**Table 2:** Statistics of annotation (a) and differentially expressed genes (b). The number of annotated unigenes is provided for each database and for each species. PL: *P. longirostris*; PM: *P. macrodactylus*.

Database	Nr		Nt		Swiss-Prot		KEGG		COG		GO	
Species	PL	PM	PL	PM	PL	PM	PL	PM	PL	PM	PL	PM
(a)												
Number of unigenes	24,547	13,469	8,243	5,416	20,974	11,608	18,194	10,232	9,183	5,161	11,490	6,635
**(b)**												
Number of unigenes	6,222	1,181	2,386	513	5,305	967	4,746	846	2,698	477	2637	323

**Figure 1:**
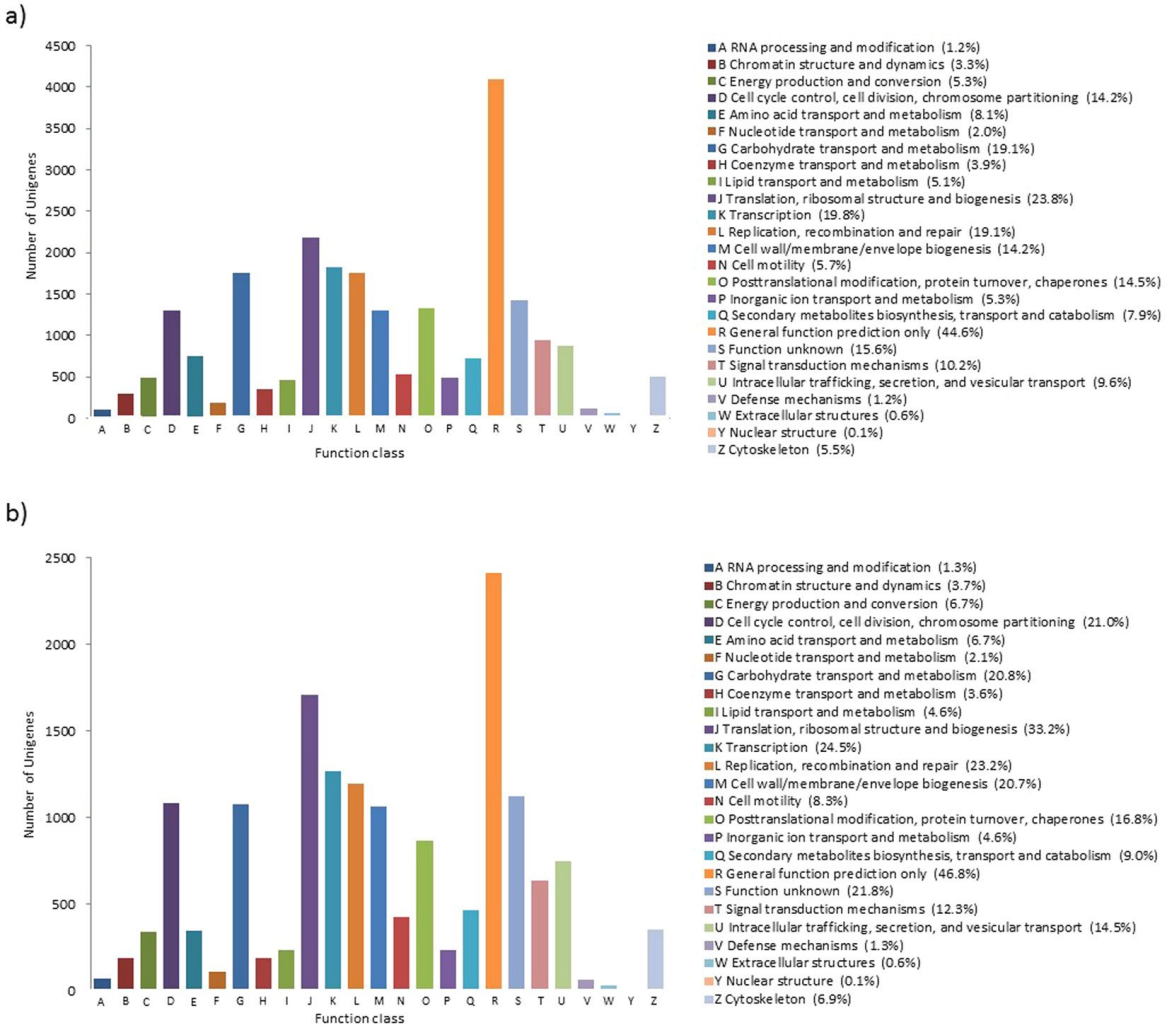
COG functional classification of all unigenes for (**a**) *P. longirostris* (PL) and (**b**) *P. macrodactylus* (PM). 9,183 (PL) and 5,161 (PM) unigenes were classified into 25 categories.

Using the COG database, 9,183 (PL) and 5,161 (PM) unigenes were classified into 25 COG categories. The first four largest functional groups were common to both species with different percentages (Fig. 1). The first category was “general function prediction only”, followed by the category “translation, ribosomal structure, and biogenesis”, the category “transcription”, and the category “replication, recombination and repair”.

**Figure 2:**
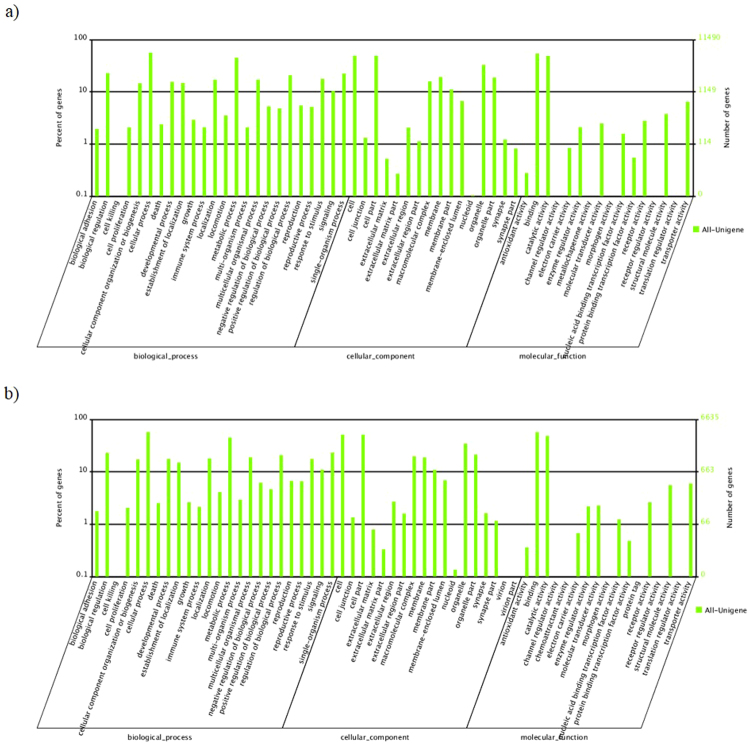
Distribution of the annotated unigenes assigned to the biological process, cellular component and molecular function for (**a**) *P. longirostris* (PL) and (**b**) *P. macrodactylus* (PM) according to the GO database. In total, 35,908 (PL) and 21,754 (PM) unigenes were assigned to the biological process category, 24,226 (PL) and 14,888 (PM) to the cellular component category, and 14,240 (PL) and 8,449 (PM) to the molecular function category.

To identify protein sequences from matches with the nr database, GO terms were assigned to the unigenes based on their Blast hits. We were able to assign 11,490 (PL) and 6,635 (PM) unigenes to at least one GO term (Table 2a), these constituting three ontologies: biological process, cellular component and molecular function (Fig. 2). Among biological processes, the cellular process was the most frequent for both species with 6,475 (PL) and 3,822 (PM) unigenes annotated. For the cell component category, cell and cell part terms were the two most abundant for both species with 5,678 (PL) and 3,400 (PM) unigenes annotated. Binding was the most abundant term represented within the molecular function category of both species (6,123 unigenes for PL and 3,797 unigenes for PM).

To understand gene function, 18,194 (PL) and 10,232 (PM) unigenes were mapped to the reference pathways recorded in the KEGG database (Table 2a). Of the 257 predicted KEGG pathways, metabolic pathways represented the largest group for both species with 13.25% unigenes annotated for PL and 11.97% for PM. The remaining unigenes were annotated to other KEGG pathways with percentages of less than 5% for the two species (Supplementary Table S1).

### Differential gene expression and enrichment analysis

**Figure 3:**
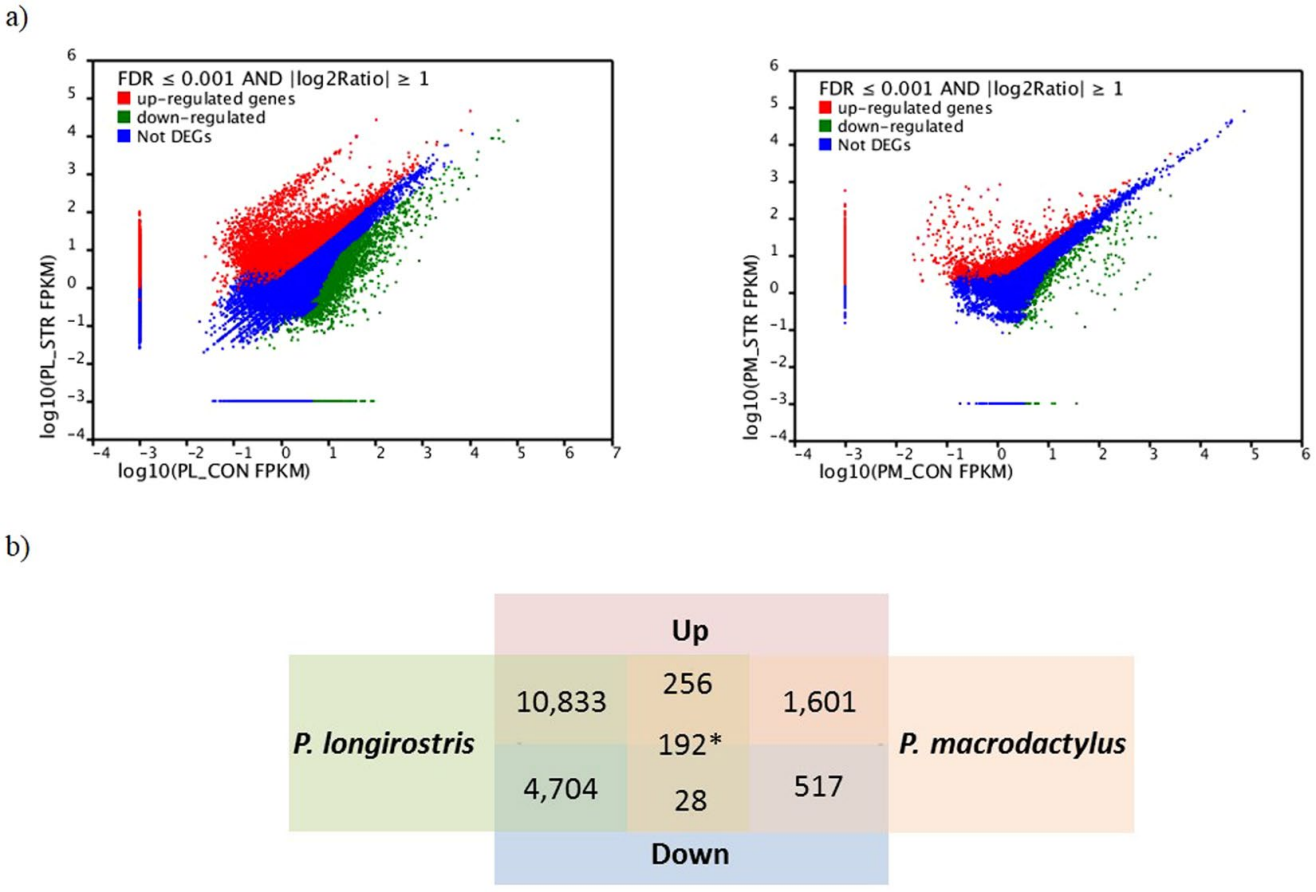
The differentially expressed genes of *P. longirostris* (PL) and *P. macrodactylus* (PM) after heat and high salinity exposure. (**a**) “Not DEGs” indicates “not differentially expressed genes”. The horizontal axis indicates the log_10_ of transcripts per million of the control and the vertical axis indicates the log_10_ of transcripts per million of the treatment. The criteria for screening differentially expressed individuals are based on FDR ≤0.001 and the absolute value of log_2_ Ratio ≥ 1. (**b**) Numbers of genes upregulated (purple) or downregulated (blue) in *P. longirostris* (green) and *P. macrodactylus* (pink), or both species (central column). *Number of differentially expressed genes changed in both species but with opposite changes in expression.

Differentially expressed unigenes (DEG) analysis was performed to identify gene expression changes in PL and PM associated with simultaneous heat and salinity exposure. The unigenes were considered as statistically significant DEGs if the false discovery false (FDR) was less than 10^−3^ and the log_2_ ratio of FPKM (fragments per kb per million fragments) values were greater than 1. A total of 16,013 genes were significantly differentially expressed in PL compared to the control treatment (Fig. 3a). This included 11,200 and 4,813 genes that were up- and down-regulated respectively (Fig. 3b). Compared to PL, PM exhibited a smaller set of genes with differentially expression levels: 2,594 genes were found to have significant changes in expression, with 1,968 genes up-regulated and 626 genes down-regulated (Fig. 3b). In order to examine the extent of similarities between the two species, we analysed the sets of DEG and identified overlap between them. A total of 294 genes were differentially expressed in both PL and PM, with 256 upregulated and 28 downregulated genes. Among common DEG, similar molecular pathways were involved: 58 and ten for the up- and downregulated genes respectively, most of them corresponding to regular cellular mechanisms (Supplementary Table S2). Surprisingly, 192 genes exhibited changes in gene expression in opposite directions (Fig. 3b; Supplementary Table S2).

For a given gene that was common to both species but with a gene expression in opposite directions, the molecular pathways were, in some cases, known for one species but not for the other, or known or unknown for both species, making further biological interpretation speculative given the scarce genomic knowledge of the studied species. Due to the different associations between up- and down-regulated genes in each species, the assigned molecular pathways for a given gene often differed between the species (Supplementary Table S2). In addition, some gene expression changes were unique to each species (Fig. 3b). For each species, more genes were found to be upregulated than downregulated, and PL exhibited almost seven times more differentially expressed genes than PM.

The DEG sequences were searched against the nr and Swiss-Prot protein databases using the blastx algorithm (Table 2b; Supplementary Table S3). For the nr database, 4,872 unigenes for PL (including 3,166 and 1,836 up- and down-regulated respectively) and 860 unigenes for PM (including 639 and 241 up- and down-regulated respectively) appeared non-redundant (nr-ID duplicates removed). Nearly 21% (PL) and 30% (PM) of blastx results had top hits against the nr database with high E-values (>10^−10^), while fewer than 33% (PL) and 16% (PM) had E-values below 10^–50^. For the Swiss-Prot database, among the 3,390 (PL) and 576 (PM) non-redundant genes, 2,349 (PL) and 434 (PM) appeared upregulated, and 1,247 (PL) and 180 (PM) downregulated. Top hits with high E-values (>10^–10^) represented nearly 15% (PL) and 22% (PM), while E-values below 10^–50^ were fewer than 32% (PL) and 11% (PM). Results of E-value top hits for both nr and Swiss-Prot databases are consistent with the relative paucity of genomic resources for crustaceans^40^. Thus, these results contrast with those from other species like the hexapod *Lygus hesperus*, which returned more than 55% of top hits with E-values below 10^–50^ ^41^.

The taxonomic distributions of top hits in the blastx results are also consistent with the under-representation of crustaceans in genomic resources. For the nr database, Arthropods appeared as the most representative taxonomic group (the branchiopod crustacean *Daphnia pulex* and the hexapod *Tribolium castaneum* being the first and second largest top hits respectively), while for the curated Swiss-Prot database, Chordates are the most represented (Supplementary Table S4).

GO and KEGG analyses were performed to identify associations between the DEGs and functional clusters and biochemical pathways. A total of 16,160 (PL) and 1,998 (PM) DEGs were assigned to terms in three categories (biological process, cellular component and molecular function) of the GO database (Supplementary Table S5). Among the biological process category, the most highly represented processes were the “primary metabolic process” and the “cellular metabolic process” for PL, and the “cellular process” and the “metabolic process” for PM. The most highly represented groups in the molecular function categories were “hydrolase activity”, “organic cyclic compound binding” and “heterocyclic compound binding” for PL, and “catalytic activity” and “binding” for PM. Under the category of cellular components, the “intracellular part”, “organelle” and “intracellular organelle” for PL, and the “cell” and “cell part” for PM were the most highly represented components. From the differentially expressed genes, 1,196, 1,432 and 929 (for PL) and 177, 216 and 124 (for PM) of the upregulated genes and 643, 742 and 532 (for PL) and 52, 61 and 50 (for PM) downregulated genes were annotated for each category (biological process, molecular function and cellular component respectively). The biological process category showed 52 (PL) and 124 (PL), and 17 (PM) and eight (PM) upregulated genes respectively for the “cellular process” and “metabolic process” GO terms. For the molecular function category, 128 genes (PL) and 23 genes (PM) were upregulated for the “binding” GO term and 81 (PL) and 16 (PM) for the “catalytic activity” GO term.

For the KEGG pathways, the most represented of the 225 pathways were metabolic pathways (16.5% genes) for PL and *Vibrio cholerae* infection (13.8% genes) for PM. Other pathways were detected for PL but at a level of representation lower than 5% (e.g. regulation of actin cytoskeleton, *Vibrio cholerae* infection, amoebiasis). Other pathways for PM were better represented (e.g. amoebiasis 13.0%, pathogenic *Escherichia coli* infection 8.3%; Supplementary Table S6).

Of the 1,672 PL gene ID’s analysed, results showed 66 enriched functional clusters formed, including 1,284 genes, from the identified DEGs. The remaining 388 genes were not clustered into significant functional groups. For PM, of the 479 gene ID’s analysed, 23 enriched functional clusters, including 249 genes, were formed from the identified DEGs. The remaining 230 genes were not clustered into significant functional groups. According to the enrichment score, the most important clusters for both species concern cuticle, keratin and chitin proteins. Heat shock protein clusters were also present but with lower enrichment scores (Supplementary Table S7). For PL, 117 DEGs mapped to direct protein-protein interactions and comprised one major subnetwork centred around FBpp0081153. This gene codes for the protein “tubulin alpha chain” from *D. melanogaster* which is known to play a role in molecular function (e.g. GTPase activity, GTP binding, structural constituent of cytoskeleton) and in biological processes (e.g. microtubule-based processes).

**Figure 4:**
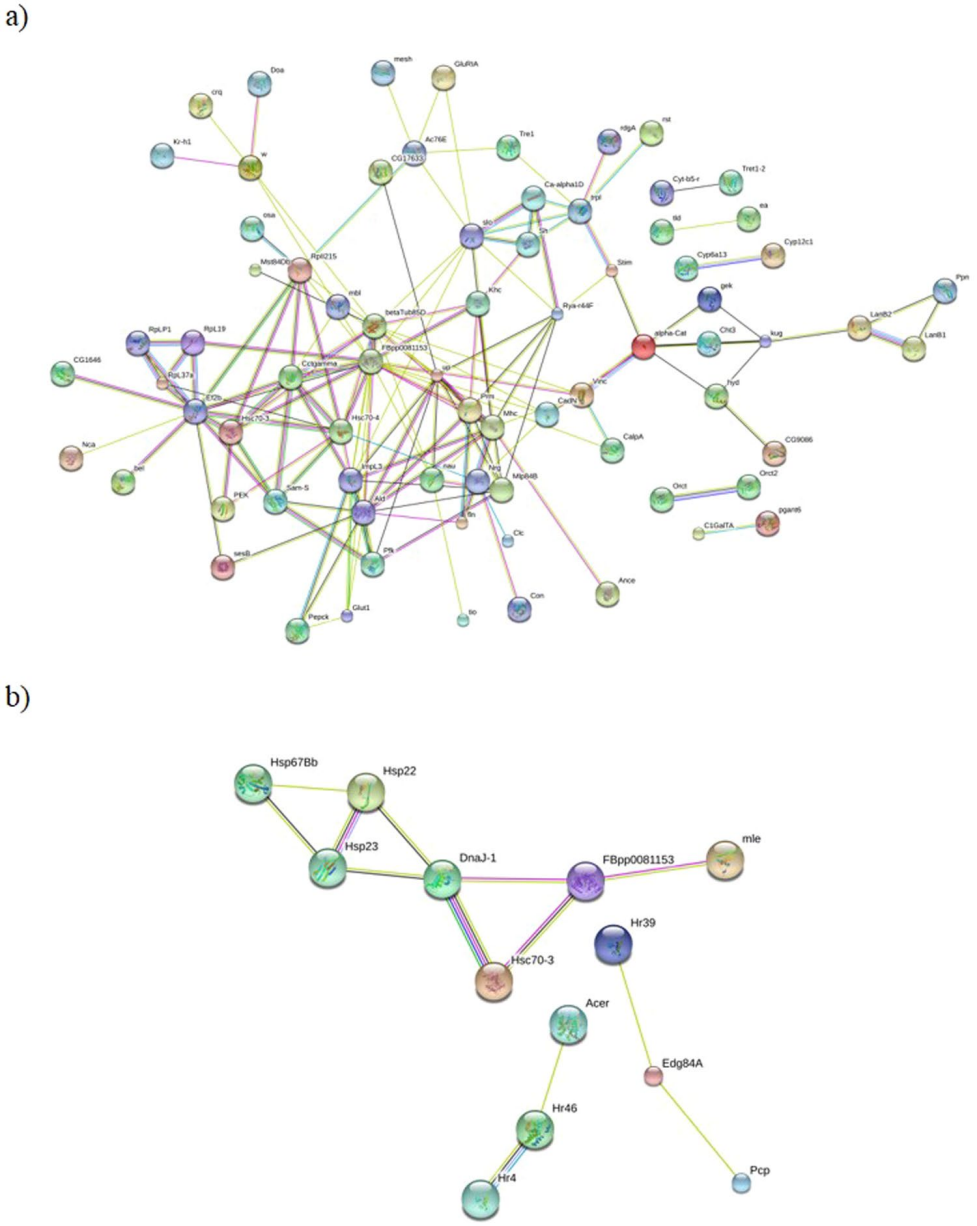
Protein-protein interaction network constructed from enriched clusters of differentially expressed genes (DEGs) for *P. longirostris* (**a**) and *P. macrodactylus* (**b**). Each node represents all the proteins produced by a single, protein-coding gene locus. Small nodes represent proteins of unknown 3D structure whereas structure is known or predicted for large nodes. Proteins jointly contribute to a shared function; this does not necessarily mean they are physically bound to each other. Network is derived from the STRING database (http://string-db.org/).

Additional simple two-way interactions were found between Cyt-b5-r (putative role in muscle cell metabolism) and Tret1–2 (function unknown); tld (required for normal dorsal development) and ea (a component of the extracellular signalling pathway that establishes the dorsal-ventral pathway of the embryo); Cyp6a13 (may be involved in the metabolism of insect hormones and in the breakdown of synthetic insecticides) and Cyp12c1 (unknown function); Orct and Orct2 (both genes probably transport organic cations); and C1GalTA (glycosyltransferase that generates the core 1 O-glycan Gal-beta1–3GalNAc-alpha1-Ser/Thr) and pgant6 (glycopeptide transferase involved in O-linked oligosaccharide biosynthesis) (Fig. 4a). FBpp0081153 was also a central node in one of the three subnetworks identified for PM. Similarly to PL, this gene was also associated with Hsc70-3 but also with other heat shock proteins and with DnaJ-1 (unknown function) and mle (required in males for dosage compensation of X chromosome linked genes). The other two subnetworks involved were: Acer (may be involved in the specific maturation or degradation of a number of bioactive peptides), Hr46 (putative receptor whose ligand is not yet known), and Hr4 (coordinates growth and maturation by mediating endocrine responses to the attainment of critical weight during larval development) together; and Hr39 (acts as a cofactor to fushi tarazu) with Edg84A and Pcp (these last two genes are for components of the cuticle of the pupa of the fruit fly) (Fig. 4b).

## Discussion

Organisms are evolutionarily constrained to develop local adaptations to cope with spatially and temporally variable environments, such as estuarine habitats. Local adaptations may arise through selection acting directly on standing genetic diversity but may also be driven by the level of transcriptomic variation available (plastic response). Thus, studying the regulation of gene expression in a variable environment is of great importance to understand the underlying molecular mechanisms of these adaptations and the relative roles of genetic and plastic responses. For the first time, the transcriptomes of the invasive shrimp *P. macrodactylus* and the native shrimp *P. longirostris* have been analysed and compared after an experiment reproducing the summer conditions (of high temperature and salinity) in the Guadalquivir Estuary, south-west Spain.

Environmental change commonly induces stress in organisms whose primary responses are changes in the level of gene expression^19,42,43^. Resulting stress levels are the results of the interactions among multiple environmental variables and their duration, intensity and frequency. However, most studies only focus on the molecular responses associated with single environmental variables and thus cannot always capture the complexity of all challenging variables. In addition, very few studies have focused so far on the comparative DEGs among sympatric and congeneric species, especially when one has an invasive status. In our study, non-redundant genes from the nr and the Swiss-Prot databases showed a large number of DEGs for both species in response to the stress induced by the combination of temperature and salinity. Such changes in gene expression levels upon stress are common and have been observed in other shrimp species such as the closely related *Palaemonetes pugio* ^44^, or the penaeid shrimp *Fenneropeneaeus chinensis* ^45^, but also in other aquatic organisms such as the redband trout *Oncorhynchus mykiss gairdneri* ^46^ or the Magadi tilapia *Alcolapia grahami* ^25^. Interestingly, in the present study the amount of DEGs varied greatly between the two *Palaemon* congeners with the invasive PM showing 5–10 fold fewer DEGs than its native congener PL when exposed to identical experimental conditions. In addition, it is notable that the common number of DEGs between the two species was very reduced compared to the DEGs unique to each species (Fig. 3), with a large fraction of common DEGs (192 of 486 total) showing opposite trends. However, when looking at the molecular pathways of each common DEG, they are surprisingly different (Supplementary Table S2). While this may seem counter-intuitive, it should be noted that genes and their products typically act in multiple pathways, and that the analysis considers the whole suite of up- and down-regulated genes within a species when assigning likely pathways. Thus, although some DEGs can be shared by the two species and expressed in opposite directions, this does not mean that the molecular pathways associated to a specific gene are the same for both. However, these interesting and unexpected results need to be interpreted with caution given the low number of samples used for both species and the large hierarchical structure of GO databases, which can lead to pathway overlap that can obscure the true source of an association signal^47^.

By matching to GO and KEGG databases, possible functions of the DEGs were analysed in order to learn more about the physiological responses of PL and PM to simultaneous thermal and salinity stress. A significant portion of the unigenes could be functionally annotated, helping to understand the adaptation process of these two species upon heat/salinity stress. For instance, the most represented KEGG pathway for PL was “metabolic pathways” (16.5% of DEGs with functional annotation), which are required for the maintenance of homeostasis^48^. Other significant KEGG pathways for PL (<5% of DEGs with functional annotation) suggest responses to pathogens (virus or bacteria) or potential cardiomyopathy (Supplementary Table S6). Conversely, the most represented KEGG pathways for PM were not homeostasis-related but were involved in the response to pathogens. Among DEGs with functional annotation, 13.8% and 13% were related to “*Vibrio cholerae*” and “amoebiasis” KEGG pathways respectively. *Vibrio* infections are very common in marine and aquatic environments, especially in arthropods^49,50^. They have been previously reported as opportunistic agents acting during heat stress, leading in some cases to massive mortalities of invertebrates such as echinoderms, cnidarians, and poriferans^51–53^. Amoebiasis is related to the *Entamoeba histolytica* parasite in human infection. It appears both species constantly respond to pathogens and parasites in their aquatic environment, and as such have evolved complex systems and signal pathways against infection, as quantified herein. Although the induction of these pathogen response pathways may likely be increased in periods of high abiotic stress, their importance differs among our native and introduced *Palaemon* species facing identical combined stress of temperature and salinity. The native species PL seems to have to face stronger physiological stress, as metabolic pathways involved in the maintenance of homeostasis are mainly activated in addition to pathogen resistance pathways. Conversely, experimental abiotic stress does not appear to directly induce specific physiological stress in PM, but might affect this introduced species through pathogen infection. These results are in accordance with previous comparative ecophysiological studies. Lejeusne *et al*.^10^ demonstrated that PM is more tolerant to a rapid increase of temperature (similar to the one used in this study) than the native congeneric and sympatric species PL. Mortality rates under acute stress conditions and oxygen consumption rates over a broad range of temperatures and salinities are also in favour of the introduced species^10^. Such differences between native and invasive species in terms of physiological plasticity have also been reported previously. For instance, Lockwood *et al*.^54^ showed that the invasive mussel species, *Mytilus galloprovincialis*, has a greater tolerance for high temperatures than the native species *M. trossulus*, which has a greater tolerance for a decrease of salinity.

In the present study, the protein-protein interaction networks also highlight the complexity of the response to increased temperature and salinity. Both species showed an increase in activity in subnetworks centred on the proteins FBpp0081153 and Hsc70–3. These subnetworks contain elements related to cytoskeleton structural support and intercellular transport. The size and complexity of this subnetwork is again much greater in the native PL than in the invasive PM, potentially indicating that the native species has set up a much more extensive response to heat and salinity stress. In response to various stressors including the strong driver of biological functions^55^ that is thermal stress, one major mechanism of molecular acclimatization is the transcriptional regulation of heat shock proteins (HSPs). The HSPs are well known protein chaperones protecting the cell from inappropriate interactions with denatured or aggregated proteins^56^, and are conserved across a large range of taxa^57^. Considering our experimental design, involving simultaneous exposure to heat and saline stress, we expected a strong enrichment of HSPs in response to an increase of temperature and salinity. They were present in enriched clusters identified in the analysis but they were not actually more important than other clusters. In fact, the most important clusters concerned cuticle, chitin and keratin, which are involved in the development and the growth of the exoskeleton, but also help by protecting cells from damage or stress^58,59^. Some studies already reported that HSP expression can be reduced or inhibited under different levels of temperature and salinity^60–63^. For example, Chen *et al*.^19^ also showed that lipids and specific fatty acids, more than HSPs, could be expressed at high to low salinity levels, modifying the gill membrane structure and controlling ion balance in the Pacific White Shrimp *Litopenaeus vannamei*. The two studied *Palaemon* species have been reported to be more sensitive (higher mortality) to high levels of salinity than to temperature^10^. This might suggest that the transcriptional up-regulation of the cuticle, chitin and keratin pathways in this study might be related to some insulation effect due to a high level of salinity, rather than an increase of temperature.

Considering the daily and seasonal increases of both temperature and salinity in the Guadalquivir Estuary, especially during summer, PM seems more able to cope with these fluctuating parameters than the native species PL. In the present context of global climate change, any increase of temperature might thus favour the invasive species and increase the physiological stress for the native PL. The future outcome in terms of competition between these species will therefore depend on evolved differences in physiological tolerances to these environmental factors in conjunction with differences in behaviours. From this perspective, the apparent enhanced ability of PM to adapt to a fluctuating environment could potentially be a strong advantage. However, our results must be treated with caution because in both species there are many genes for which we currently do not have known functional annotations, and some of these may eventually be demonstrated to play a major role in metabolism or other physiological aspects of thermal/salinity stress. Furthermore, it was not technically possible to control for other factors such as sex or age in this study, and such factors may strongly influence the comparative response of the native and introduced *Palaemon* species.

## Conclusion

Every summer, European estuaries experience a substantial increase in temperature and salinity, forcing organisms like the native shrimp PL and its invasive counterpart PM to respond phenotypically in the short term so as to cope with the environmental stress imposed by seasonal variations. This study constitutes the first investigation of the transcriptome response of these two species to a simultaneous increase of temperature and salinity. Our results provide preliminary but new insights into the molecular mechanisms involved in the response to multiple abiotic stress factors by congeneric species. It is a rare study comparing two closely related species with different invasive status, and suggests that the scale of the plastic response appears much greater in the invasive species compared to the native species.

## Methods

### Shrimp collection

Individuals of the native shrimp PL and the oriental shrimp PM were sampled at La Esperraguera in the Guadalquivir estuary, southwest Spain. Living individuals were collected in September 16^th^, 2011 using shrimp keep-nets (mesh-size 4 mm) placed at low tide and recovered 24 h later. Species were identified *in situ* then transported live to the laboratory within one hour. In order to reduce catching and manipulation stress, living shrimps were acclimated during 48 h before the experiment in aerated aquaria with artificial saltwater at 20 °C and a salinity of 5 ppt, obtained by dissolving dry sea-salt Instant Ocean (Aquarium Systems, Mentor, Ohio) in distilled water. These are the usual conditions found in the Guadalquivir estuary during the spring at low tide. Salinity was measured using the Practical Salinity Scale. Aquariums were placed in a climatic chamber (Fitoclima 10000 EHHF, Aralab) on a 12 h:12 h dark:light photoperiod. Shrimps were fed daily *ad libitum* with commercial aquarium food (gammarids) before the experiment.

### Stress experiment

After acclimation, 20 individual shrimps of each species were placed individually in small, closed plastic aquaria (0.35 L) with a 1mm mesh sieve at the bottom and placed within 91 L experimental aquaria at 20 °C and S = 5 ppt (control condition). The stress condition was conducted on 10 shrimps for each species placed for 1 h in another 91 L aquarium at 30 °C and S = 15 ppt, before being placed back into the control aquarium for one additional hour. The experiment was then stopped and all individuals were immediately frozen in liquid nitrogen then stored at −80 °C before RNA extraction.

### Sample preparation for RNA-Seq

Total RNA was extracted from the hepatopancreas or midgut gland of three individuals of PL and five of PM from each of the experimental and control groups using TRIzol reagent (LifeTechnologies) according to manufacturer’s instructions and treated with DNase I to remove genomic DNA. The low number of samples per experimental group are typical of transcriptome characterisation studies that compared multiple environmental stressors (e.g.^25^). Individuals for each species were then pooled for samples from the treatment and control groups respectively. RNA-seq library preparation and sequencing was carried out on pools by Beijing Genomics Institute BGI (Hong-Kong, China). Briefly, mRNA was purified using oligo (dT) magnetic beads, followed by fragmentation. The cleaved RNA fragments were then used for first-strand cDNA synthesis. Short fragments were purified and resolved with EB buffer for end reparation and single nucleotide A (adenine) addition. Adaptors were ligated to the short fragments and they were selected for PCR amplification as templates. During the quality control steps, an Agilent 2100 Bioanalyzer and ABI StepOnePlus Real-Time PCR System were used to quantify and quality check the sample library. The cDNA library was sequenced from both 5′ to 3′ ends (paired-end 100 bp) using the Illumina HiSeq^TM^ 2000 platform.

### *De novo* assembly of transcriptome

Raw data was pre-processed before *de novo* assembly to remove reads containing more than 5% of unknown or low quality bases (we defined nucleotides with a quality score less than 20 as low-quality nucleotides). *De novo* assembly of the filtered dataset was performed using Trinity (release 20121005;^64^ using the following settings:–seqType fq–min_contig_length 100–group_pairs_distance 250–path_reinforcement_distance 85–min_kmer_cov 2).

To remove redundancy in the contigs, TGICL^65^ was used to assemble all the unigenes from different samples to form a single set of non-redundant transcript fragments (termed unigenes). Unigenes showing significant similarities (up to 70%) were grouped as clusters, whereas those not having similar matches in the assembly were reported as singletons.

### Gene annotation and classification

Functional annotation of unigenes was performed using BLAST (release v2.2.26;^66^) for both species. The program Blastx allowed comparison of all unigene sequences with sequences from the nr, Swiss-Prot, the COG and the KEGG databases. The following command-line settings were used: -F F –e 1e-5 –p, with a significant threshold of E-value ≤10^–5^. The same parameters were also employed to align unigene sequences to the nt database using Blastn. A priority order of nr, Swiss-Prot, KEGG and COG was followed to align unigenes to protein databases to avoid conflict between databases. Thus, unigenes aligned to a higher priority database were not aligned to a lower priority database. When a unigene happened to be unaligned to any of the above databases, the ESTScan software (release 3.0.2;^67^) was used to predict potential coding regions and sequence directions. After the assignment process, the Gene Ontology functional classification, based on the results of nr annotation, was obtained using Blast2GO (release 2.5.0;^68^) with default parameters. This analysis allowed the mapping of all annotated unigenes to GO terms in the database, and thus the calculation of the number of unigenes associated with each GO term category: biological process, cellular component and molecular function.

### Differentially expressed genes and enrichment analysis

Reads were normalized according to FPKM in order to identify differentially expressed genes. This method allows the elimination of the influence of different gene length and sequencing depth on the calculation of gene expression. The criteria applied to determine significant DEGs included a false discovery rate (FDR) to correct the *P*-value for multiple comparisons. Here, FDR was set to less than 10^–3^ and the FPKM value cut-off was at least a twofold difference between the two sample groups. To identify well represented GO terms and KEGG pathways in DEGs compared to the genomic background, analyses were conducted by hypergeometric distribution using the program GO-TermFinder (release 0.86;^69^) and Path_Finder (unpublished software), respectively. GO terms and KEGG pathway were accepted as significantly enriched when the corrected p-value, after Bonferroni correction, was ≤0.05. We performed further functional enrichment analysis and clustering of the DEGs based on biological process and molecular function ontologies using DAVID^70,71^. Finally, we identified potential protein-protein interactions within the functional DEG clusters by querying the STRING database (v10; accessed July 5, 2016)^72,73^ using *Drosophila melanogaster* as the closest well-annotated reference.

## Electronic supplementary material

Supplementary Figures

Supplementary Dataset 1

Supplementary Dataset 2

Supplementary Dataset 3

Supplementary Dataset 4

Supplementary Dataset 5

Supplementary Dataset 6

Supplementary Dataset 7
