# Supplementary material for: Transcriptomic response to thermal and salinity stress in introduced and native sympatric Palaemon caridean shrimps
Source: Sci Rep. 2017 Oct 25;7:13980. doi: 10.1038/s41598-017-13631-6 (PMC5656633; doi:10.1038/s41598-017-13631-6)
Supplement: Supplementary file 1 — Supplementary Figures [file 41598_2017_13631_MOESM1_ESM.pdf]

**Transcriptomic response to thermal and salinity stress in introduced and native sympatric *Palaemon* caridean shrimps**

Amandine D. Marie, Steve Smith, Andy J. Green, Ciro Rico and Christophe Lejeusne

a)

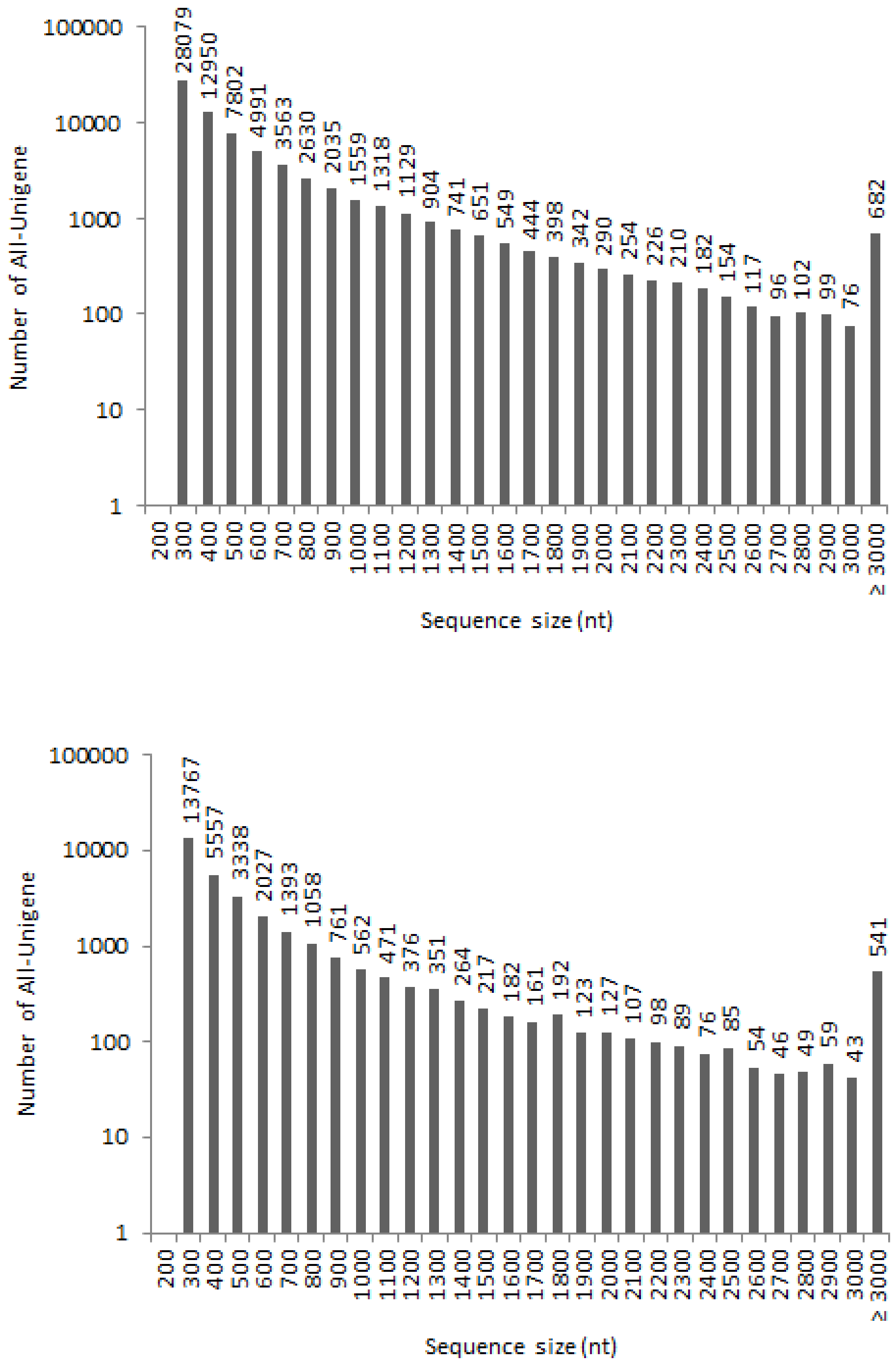


Supplementary Figure S1: Length distribution of all unigenes. The length of the unigenes for a) *P. longirostris* and b) *P. macrodactylus* ranged from 300 bp to more than 3,000 bp. The total number of unigenes within each range is given on the top of each column.

a)

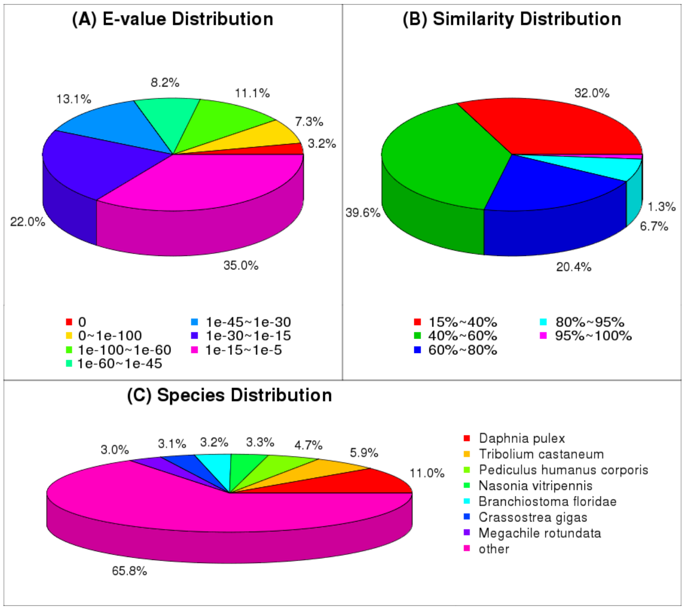


b)

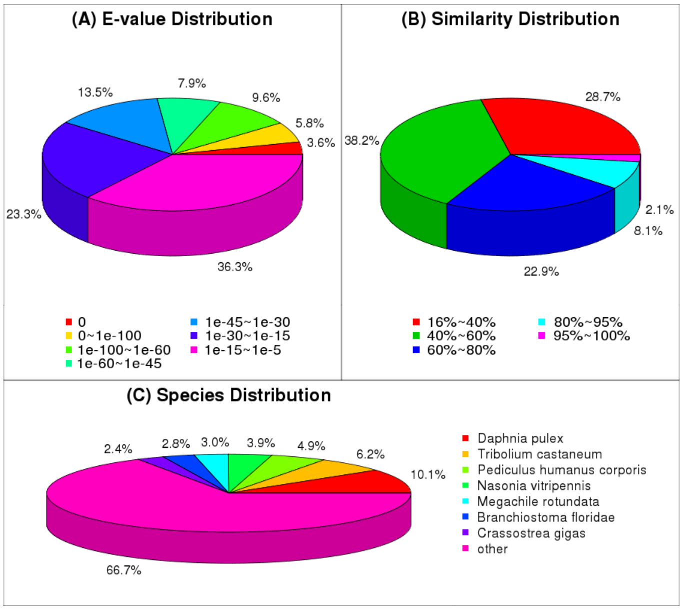


Supplementary Figure S2: E-value, similarity and species distribution in Nr annotation for a) *P. longirostris* (PL) and b) *P. macrodactylus* (PM). A) 65.0% (PL) and 63.7% (PM) of the unigenes displayed a strong homology (E-value ≥ 1.0E-15) to known unigenes; B) 68.0% (PL) and 71.3% (PM) of the unigenes showed more than 40% similarity to known unigenes; C) according to the known unigenes, the most represented species was *Daphnia pulex* (11.0% for PL and 10.1% for PM).
